# Methotrexate-Induced Toxicity After Ultrasound-Guided Intragestational Injection in a Patient with Caesarean Scar Pregnancy—A Case Report

**DOI:** 10.3390/medicina60111900

**Published:** 2024-11-20

**Authors:** Sofoklis Stavros, Anastasios Potiris, Angeliki Gerede, Athanasios Zikopoulos, Maria Giourga, Christina Karasmani, Athanasios Karpouzos, Theodoros Karampitsakos, Spyridon Topis, Ismini Anagnostaki, Konstantinos Louis, Ioannis Tsakiridis, Themistoklis Dagklis, Peter Drakakis, Ekaterini Domali

**Affiliations:** 1 Third Department of Obstetrics and Gynecology, University General Hospital “ATTIKON”, Medical School, National and Kapodistrian University of Athens, 124 62 Athens, Greece; sfstavrou@med.uoa.gr (S.S.); thanzik92@gmail.com (A.Z.); theokarampitsakos@hotmail.com (T.K.); spyrostopis@med.uoa.gr (S.T.); kostaslouisss@gmail.com (K.L.); pdrakakis@med.uoa.gr (P.D.); 2 Department of Obstetrics and Gynecology, Democritus University of Thrace, 691 00 Campus, Greece; agerede@otenet.gr; 3 First Department of Obstetrics and Gynecology, Alexandra Hospital, Medical School, National and Kapodistrian University of Athens, 115 28 Athens, Greece; giourgam@med.uoa.gr (M.G.); ckarasmani@gmail.com (C.K.); thanoskarpouzosdr@hotmail.com (A.K.); kdomali@yahoo.fr (E.D.); 4 Third Department of Obstetrics and Gynecology, General Hospital Ippokratio, Medical School, Aristotle University of Thessaloniki, 546 42 Thessaloniki, Greecetdagklis@gmail.com (T.D.)

**Keywords:** ectopic pregnancy, cesarean scar pregnancy, methotrexate, early pregnancy, pancytopenia, intragestational injection

## Abstract

*Background and Objectives*: Caesarean scar pregnancy (CSP) is a rare form of ectopic pregnancy in which the early pregnancy implants at the site of the uterine scar. Methotrexate (MTX) in lower doses can be used to treat CSPs. However, MTX administration is associated with a spectrum of side effects that include hematological toxicities. This case report presents a CSP treated with an intragestational injection of MTX and subsequently developed pancytopenia. *Materials and Methods*: A 23-year-old woman at six weeks and six days of pregnancy was referred as a potential case of CSP. After establishing the diagnosis, she was treated with a transvaginal ultrasound-guided intragestational administration of 80 mg MTX (adjusted to 50 mg/m^2^ body surface area) under sedation. *Results*: On day four after the MTX injection, she developed oral ulcers, fever, and pruritic phlyctenular maculopapular rash. Subsequently, the patient developed febrile neutropenia and was admitted to the Intensive Care Unit. On day six, a subsequent exacerbation of the rash was observed with the formation of blisters and purplish spots with concurrent odynophagia and sialorrhea. Ultimately, the patient developed pancytopenia due to bone marrow suppression. Fifteen days after MTX administration, the patient recovered and was discharged from the hospital hemodynamically stable, afebrile, with dropping β-hcg levels, and in good clinical condition. *Conclusions*: Although methotrexate administration is the preferred option for the treatment of cesarean scar pregnancies, clinicians should be aware of the fact that its use entails potential risks, even when it is used locally. To our knowledge, this case is the first description of pancytopenia due to bone marrow suppression following a single low dose of intragestational methotrexate injection.

## 1. Introduction

Ectopic pregnancy occurs when a fertilized ovum implants outside the uterine cavity. Its incidence is estimated to be around 1–2% of all pregnancies, and the most common implantation spot is the fallopian tubes [1]. Caesarean scar pregnancy (CSP) is a rare form of ectopic pregnancy in which the early pregnancy implants at the site of the uterine scar of a previous cesarean section [2]. The incidence of CSP is estimated to be about 1 in 1800 to 2500 pregnancies [3]. CSP is a challenging gynecologic high-risk condition due to its rarity, its difficulty to diagnose with a high misdiagnosis rate, and, ultimately, its severe complications (excessive bleeding, uterine rupture, and fecundity impairment) if it is not diagnosed and treated early and promptly [2,4].

Treatment options for CSP include medical management primarily with methotrexate and excisional treatments [5]. Methotrexate (MTX) is a pharmaceutical agent that inhibits dihydrofolate reductase (DHFR), a pivotal enzyme within the folic acid metabolism pathway [6]. Its application encompasses high doses for specific hematologic malignancies and lower doses for the management of autoimmune disorders, as well as the treatment of ectopic pregnancies, such as cesarean scar pregnancies [7]. Despite its proven efficacy, MTX is associated with a spectrum of potential side effects that include hematological, hepatic, renal, dermatologic, and gastrointestinal toxicities. Although these adverse effects are conventionally present at higher dosages, their occurrence remains possible even with lower-dose MTX administration [8].

In cases where methotrexate is insufficient or contraindicated, more invasive techniques are employed. These include either the evacuation of the pregnancy using suction or hysteroscopic resection, or the excision of the pregnancy with a laparotomic or laparoscopic approach [4,9,10]. Suction is the most frequently utilized invasive technique with or without additional hemostatic measures such as cervical cerclage, uterine artery embolization, and intra-uterine catheter insertion [11,12,13,14]. On the other hand, excisional techniques are more invasive and difficult to perform, but they bear the advantage of repairing the scar itself [5,15]. However, it is still unknown whether the repair of the scar is associated with a reduced risk for recurrence in future pregnancies.

This case report presents the unique case of a 23-year-old woman who was treated with an ultrasound-guided intragestational injection of MTX for a cesarean scar pregnancy and subsequently developed pancytopenia. To our knowledge, this is the first case in the literature of pancytopenia after the intragestational administration of MTX. Clinicians should be aware and alarmed that severe side effects can occur even with this route of administration with catastrophic consequences if they are not identified and treated early.

## 2. Detailed Case Description

**Figure 1:**
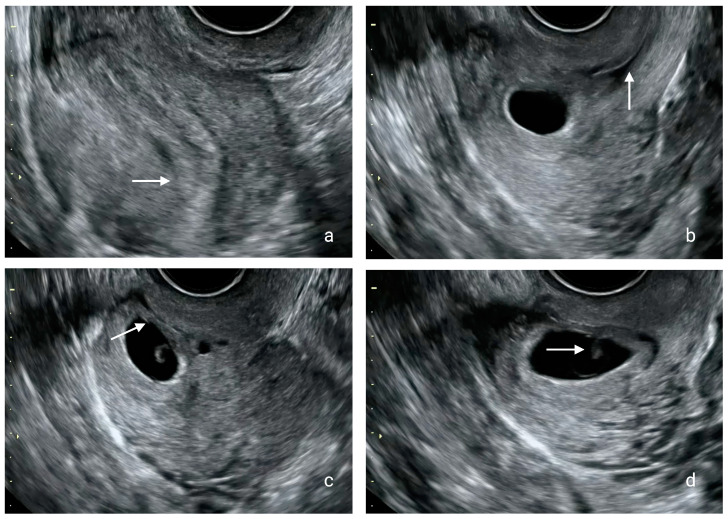
Transvaginal ultrasound findings. (**a**) The empty uterine cavity marked with a white arrow; (**b**) the empty cervical canal marked with a white arrow; (**c**) the gestational sac embedded at the site of the previous lower uterine segment and the thin layer of myometrium between the gestational sac and the bladder marked with a white arrow; (**d**) the presence of the fetal pole.

A 23-year-old G6P3 woman at six weeks and six days of pregnancy was referred to our Early Pregnancy Unit as a potential case of a cesarean scar pregnancy. The patient’s obstetrical history included three cesarean sections and two first trimester abortions treated with expectant management. The patient reported no other comorbidities, allergies, or previous operations. The patient’s height was 165 cm, their body weight was 56 kg, and their measured body surface area was 1.6 m^2^. In our department, the diagnosis was confirmed according to the diagnostic criteria [16]. Transvaginal ultrasound findings showed an empty uterine cavity with a gestational sac of 25 mm in diameter with a fetal pole (crown rump length of 0.62 cm) and positive fetal cardiac activity located anteriorly at the level of the internal os embedded at the site of the previous lower uterine segment cesarean section scar. Moreover, an empty endocervical canal and a thin layer under 2 mm of myometrium between the gestational sac and the bladder were also illustrated in the same scan (Figure 1 and Videos S1 and S2).

The patient’s beta human chorionic gonadotropin (β-hcg) levels were at 30.420 mIU/mL. Pre-treatment diagnostic analyses (white blood cells: 8.2 × 10^9^/L; hematocrit: 36%; hemoglobin: 13.5 g/dL; platelet count: 180 × 10^9^/L) were in a normal range. Subsequently, a transvaginal ultrasound-guided intragestational injection of 80 mg MTX (adjusted to 50 mg/m^2^ body surface area) was administered to the patient under sedation (Videos S3 and S4).

However, on day four after the MTX injection and while the patient was still hospitalized, she developed oral ulcers, fever (38.4 °C), and pruritic phlyctenular maculopapular rash. On day five, the clinical status of the patient remained unchanged, but the hematological assessment revealed febrile neutropenia (WBC: 0.8 × 10^9^/L; neutrophils: 35%; Hb: 11.9 g/dL; PLTs: 248 × 10^9^/L). Empiric broad-spectrum antibiotic therapy, intravenous hydration, and folate supplementation were initiated. Given the previously normal blood counts, methotrexate-induced neutropenia was suspected. The patient was admitted to the Intensive Care Unit to ensure optimal care. On day six, a subsequent exacerbation of the rash was observed, causing blisters and purplish spots to form. The patient complained of odynophagia and sialorrhea. An examination of the oral cavity revealed the erosion of the mucosal lining in both the oral and pharyngeal regions. Further drops in neutrophils (WBC: 0.4 × 10^9^/L; neutrophils 25%), anemia (HB: 7 g/dL; HCT: 22%), and thrombocytopenia (PLTs: 110 × 10^9^/L) were reported. As a rescue therapy, leucovorin (120 mg/day) was initiated, and the patient received a transfusion of platelets and packed RBCs (red blood cells) because of pancytopenia. A personalized genetic test detected Methylene Tetrahydrofolate Reductase (MTHFR) C677T polymorphism, thus indicating the decreased activity of the MTHFR [17]. On day seven, she developed further pancytopenia (WBC: 0.3 × 10^9^/L; HB: 6.8 g/dL; HCT: 21%; PLTs: 30 × 10^9^/L) and severe alopecia. Blood and urine cultures were negative. A bone marrow biopsy was performed, which revealed bone marrow suppression. She received a granulocyte colony-stimulating factor agent (filgrastim). The transvaginal ultrasound revealed a persistent gestational sac and no fetal heart rate (Video S5). On the seventh day from MTX administration, a dilation and aspiration of the sac, guided by ultrasound, was performed under anesthesia to eliminate the risk of infection.

Ultimately, 15 days after MTX administration, the patient’s hematologic parameters were normalized and the patient was discharged from the hospital hemodynamically stable, afebrile, with dropping β-hcg levels and in good clinical condition. Table 1 presents the complete timeline of the patient’s symptoms and β-hcg levels during the course of the hospitalization.

All procedures performed in this study were in accordance with the ethical standards of the institutional and/or national research committee(s) and with the Helsinki Declaration (as revised in 2013). Written informed consent was obtained from the patient for the publication of this case report and accompanying videos. A copy of the written consent is available for review by the Editorial Office of this journal.

## 3. Discussion

The prevalence of cesarean scar pregnancy is escalating due to the increased use of early gestational ultrasound and the increased rate of cesarean section deliveries [18]. As a rare form of ectopic pregnancy, CSP is a potential cause of significant morbidity and mortality in the first trimester of pregnancy. As most women affected by CSP are in their reproductive years and express a desire to preserve fertility, the focus of treatment should be on treating CSP while maintaining the woman’s reproductive potential. In this context, preference is given to medical or minimally invasive interventions over surgical approaches. Consequently, various treatment modalities have been suggested and studied [19].

Methotrexate (MTX) acts as a folic acid antagonist, and its adverse effects stem from inhibiting the enzyme dihydrofolate reductase, which is critical in purine synthesis and cell division. Consequently, tissues undergoing rapid proliferation, such as skin, bone marrow, and oral and gastrointestinal mucosa, are particularly susceptible, leading to earlier and more frequent manifestations of adverse effects. Adverse effects, such as stomatitis, leukopenia, nausea, abdominal distress, malaise, fatigue, chills, alopecia, fever, and dizziness, are reported more frequently. Additionally, the rarely occurring yet life-threatening complications of MTX treatment include myelosuppression, renal failure, severe infections, and neurotoxicity [20].

Local and systemic methotrexate therapies represent CSP’s two most extensively used treatment choices [2,3,4,7]. However, the use of systemic MTX treatment for CSP has been associated with various concerns. A recent comprehensive review revealed that nearly one-fifth of women (19%) subjected to this approach experienced significant complications, requiring additional surgery with dilation and curettage (D&C) in 99% of cases [19]. The administration of intragestational MTX injection is a widely favored therapeutic approach for CSP due to its fast response and relatively low rate of side effects [21]. However, it should be noted that the optimal dose of MTX for intragestational administration has not yet been established. Most cases adhere to the optimal dose of 50 mg per m^2^. Recent research has also suggested the addition of mifepristone to reduce the necessary dose of MTX [22].

A literature search was conducted and revealed only five cases of pancytopenia after a single dose of MTX in patients with ectopic pregnancy (Table 2). However, the administration in all five cases was systemic (either intravenous or intramuscular), and none of them involved the intragestational administration of MTX, as in our case. In three of these cases [23,24,25], MTX was administered intramuscularly, and in the other two [26,27], it was administered intravenously. Two of the patients died due to complications [25,27], and the other three recovered within 14 to 16 days after the administration of MTX [23,24,26]. In our case, after a single dose of intragestational administration of MTX, the patient developed stomatitis, oral ulcers, alopecia, skin rash, and eventually pancytopenia. All the above complications are mainly reported in cases of high-dose MTX used in oncology and rheumatology, but also in some cases of systemic administration in the treatment of ectopic pregnancy in gynecology. It is extremely rare to face this kind of side effect when using MTX locally. Genetic testing also revealed that our patient had a genetic mutation in the MTHFR. One of the most frequently occurring single nucleotide polymorphisms in the MTHFR gene is C677T, indicating a reduced enzyme activity. It is known that the MTHFR polymorphism could act as a predictor of MTX toxicity in rheumatoid arthritis patients [28].

## 4. Conclusions

Although methotrexate is a very useful drug regimen in the treatment of cesarean scar pregnancies, clinicians should be aware that its use entails potential risks, even when it is used locally. To our knowledge, this case represents the first description of pancytopenia due to bone marrow suppression following a single dose of intragestational methotrexate injection for the treatment of a cesarean scar pregnancy. Further study is essential in the management of these complex cases to achieve the best possible outcome with minimally invasive and fertility-sparing treatments.

## Figures and Tables

**Table 1:** Follow-up with beta HCG and the patient’s symptoms after the administration of intragestational methotrexate.

Day (After MTX Administration)	β-HCG Levels (I.U.)	Symptoms
0 (day of administration)	56.529	No symptoms
1	65.595	Mild vaginal bleeding
2	53.860	Mild vaginal bleeding
4	43.845	Fever, pruritic skin rash, sore throat
6	23.573	Fever and pruritic skin rash, sore throat, mucositis, odynophagia, sialorrhea, neutropenia
8	17,673	Pruritic skin rash, sore throat, mucositis, odynophagia, alopecia, pancytopenia
11	3755	Pruritic skin rash, sore throat, mucositis, odynophagia, alopecia, pancytopenia
13	655	Pruritic skin rash, sore throat, mucositis, odynophagia, alopecia
14	300	Skin lesions and mucositis on recovery, normal blood tests
16	140	Skin lesions and mucositis on recovery, normal blood tests
17	71.8	Skin lesions and mucositis on recovery, normal blood tests
19	8	Normal blood tests, skin lesions and mucositis healed.

**Table 2:** Cases in the literature with pancytopenia after a single low dose of methotrexate.

Study	Age (in Years)	History	Methotrexate Dosage	Initial Symptoms	Outcome
Isaac Jr et al. [23]	23	Previous ectopic pregnancy with left salpingectomy	50 mg/m^2^ i.m.	Vomiting, mucositis, fever, and pruritic rash	Recovery from day 14
Shao S. et al. [24]	27	No comorbidities	50 mg i.m.	Vomiting, mucositis, fever, and skin rash	Recovery from day 16
Gaies E. et al. [25]	32	Penicillin allergy	44 mg/m^2^ i.m.	Mucositis, odynophagia, dysphagia, fever, and skin lesions	Deceased (pancytopenia, septic shock, and hepatic cytolysis)
Willner et al. [26]	21	No comorbidities	100 mg i.v.	Sore throat, skin rash, and fever	Recovery from day 15
Kelly et al. [27]	N/A	Hemodialysis-dependent (renal failure)	50 mg/m^2^ i.v.	Abdominal discomfort, nausea, vomiting, and severe mouth pain	Deceased (pancytopenia, acute respiratory distress, and bowel ischemia)
Present Case	23	No comorbidities	80 mg intragestational injection	Mucositis, odynophagia, fever, and skin rash	Recovery from day 15

## Data Availability

The data that support the findings of this study are available from the corresponding author upon reasonable request.

## Supplementary Materials

The following supporting information can be downloaded at: https://www.mdpi.com/article/10.3390/medicina60111900/s1, Video S1: The endometrial cavity is empty, but the gestational sac is clearly visible. Video S2: The sac contains a yolk sac and pole with a positive fetal heart rate. The cervical canal is also closed. Video S3: The needle is delicately guided into the endometrial cavity, revealed by transvaginal ultrasonography, and then followed by gentle fluid drainage. Video S4: The MTX is administered directly into the endometrial cavity. Video S5: The yolk sac and the fetal pole are no longer visible in the gestational sac the following day.
